# Efficacy and safety of third-line or later-line targeted treatment for patients with metastatic colorectal cancer: a meta-analysis

**DOI:** 10.3389/fonc.2023.1165040

**Published:** 2023-05-31

**Authors:** Wen-Hui Xue, Xue-Wei Li, Ya-Qian Ding, Na Wu, Bei-Bei Pei, Xiao-Yan Ma, Jun Xie, Wen-Hui Yang

**Affiliations:** ^1^ Department of Digestive Oncology, Cancer Center, Shanxi Bethune Hospital, Shanxi Academy of Medical Sciences, Tongji Shanxi Hospital, Third Hospital of Shanxi Medical University, Taiyuan, Shanxi, China; ^2^ Department of Biochemistry and Molecular Biology, Shanxi Key Laboratory of Birth Defect and Cell Regeneration, Shanxi Medical University, Taiyuan, Shanxi, China; ^3^ Department of Gastroenterology, Shanxi Province Cancer Hospital/Shanxi Hospital Affiliated to Cancer Hospital, Chinese Academy of Medical Sciences/Cancer Hospital Affiliated to Shanxi Medical University, Taiyuan, Shanxi, China

**Keywords:** efficacy, safety, metastatic colorectal cancer, meta-analysis, targeted therapy

## Abstract

Targeted therapy has been standardized in front-line therapies for metastatic colorectal cancer (mCRC), while explicit recommendations for third- or later-line are still lacking. This study evaluated the efficacy and safety of combining targeted therapy with chemotherapy in the third- or later-line treatment for mCRC *via* meta-analysis, providing evidence-based guidance for clinical or research practice. Comprehensive retrieval of related studies was conducted according to the PRISMA guideline. Studies were stratified with patient characteristics and pharmacological classification of the drugs. For the data available for quantitative analysis, pooled overall response rate, disease control rate, hazard ratios (HRs) for overall survival (OS) and progression-free survival (PFS), and adverse events rate with respective 95% confidence intervals (CIs) were calculated. A total of 22 studies (1,866 patients) were included in this meta-analysis. Data from 17 studies (1,769 patients) involving targets of epidermal growth factor receptor (EGFR) and vascular endothelial growth factor (VEGF) were extracted for meta-analyses. The overall response rates for monotherapy and combined therapy were 4% (95% CI: 3%, 5%) and 20% (95% CI: 11%, 29%). The pooled HRs (combined therapy *vs.* mono) for OS and PFS were 0.72 (95% CI: 0.53, 0.99) and 0.34 (95% CI: 0.26, 0.45). Another five studies were included in narrative depiction, involving targets of BRAF, HER-2, ROS1, and NTRK. The findings of this meta-analysis indicate that VEGF and EGFR inhibitors manifest promising clinical response rates and prolonged survival in the treatment of mCRC with acceptable adverse events.

## Introduction

Colorectal cancer (CRC) is one of the most common malignancies worldwide; the estimated annual incidence and mortality are 19.7/100,000 and 8.9/100,000 (1, 2). Among patients diagnosed with CRC, 20% had metastatic colorectal cancer (mCRC) and 40% had recurrence after previous treatment of localized diseases (3, 4). Furthermore, prognosis remains poor after standard treatment for patients with mCRC, with a median 5-year survival rate of less than 20% (4).

At present, the standard first-line and second-line therapies for mCRC are a combination of doublet or cytotoxic triplet chemotherapy and targeted therapies, including anti-epidermal growth factor receptor (EGFR) or anti-vascular endothelial growth factor (VEGF) antibody, the choice of treatment is influenced by patient features, cancer characteristics, and molecular profiles (5–8). In addition, RAS and BRAF tests are recommended by the European Society for Medical Oncology (ESMO) and the United States (US) National Comprehensive Cancer Network (NCCN) guidelines before the initiation of first-line therapy (9, 10). The choice of second-line regimen depends on the first-line systemic treatment, and approximately two-thirds of mCRC patients received second-line treatment (11). Fluorouracil, folinic acid, and irinotecan (FOLFIRI) and fluorouracil, folinic acid, and oxaliplatin (FOLFOX) are typical second-line chemotherapy options for mCRC patients (12). However, the efficacy of chemotherapy is very low in the third-line treatment of CRC, and tumor shrinkage is rarely observed (13). Immunotherapy revolutionized the oncology landscape in the past 10 years, pembrolizumab or nivolumab are recommended as treatment options in second-line and beyond for patients with deficient MMR/MSI-high mCRC (11, 12). For CRC patients receiving third-line treatment, considering molecular cancer characteristics and clinical trial registration is an important aspect of management (12). Cetuximab or panitumumab is particularly effective for KRAS/NRAS wild-type mCRC patients not previously treated with EGFR antibodies and is recommended as the standard treatment for the third-line or later-line follow-up treatment (14, 15). Regorafenib is recommended in RAS wild-type patients previously treated with EGFR antibodies (10). Furthermore, receptor tyrosine kinase inhibitor (rTKI) has been shown to prolong progression-free survival (PFS) in refractory mCRC patients with acceptable tolerability (16). Agents targeting human epidermal growth factor receptor-2 (HER2), neurotrophic tyrosine receptor kinase (NTRK), and c-ros oncogene 1, receptor tyrosine kinase (ROS1) were used in the treatment of mCRC (17–19). Nevertheless, EGFR inhibitors are associated with toxicity, including rash and diarrhea in tissues expressing EGFR. Multi-kinase inhibitors can cause hand-foot skin reactions, rash, fatigue, diarrhea, and hypertension (20). Therefore, when the quality of life gains importance as a therapeutic goal, the difference in the mechanism of action and, more importantly, the safety of available third-line/later-line mCRC therapy may guide the treatment choices of individual patients.

Targeted therapy has been standardized in front-line therapies for mCRC, but explicit recommendations for third- or later-line are still lacking. As far as it is concerned, several studies reported the efficacy and safety of targeted treatment alone or combined chemotherapy (16, 21–28). This study aimed to conduct a meta-analysis through a synthesis of the evidence to generate a comprehensive assessment of efficacy and safety of third-line or later-line targeted treatment for patients with mCRC and subsequently to provide evidence and clues for clinical research and practice.

## Materials and methods

### Statements

This meta-analysis was conducted based on published citations that had declared ethical approvals, and no original clinical raw data of the published results were collected or utilized, thereby ethical approval was not warranted for this study. This study was based on the Preferred Reporting Items for Systematic Reviews and Meta-analysis (PRISMA) (29).

### Search strategy and selection criteria

We systematically searched the online electronic databases, PubMed, Scopus, and Embase, from the databases’ inception to June 16, 2022, with articles in English all considered. The following keywords and terms were used for the online database search: third-line, later-line, fruquintinib, famitinib, bevacizumab, ramucirumab, cetuximab, panitumumab, trastuzumab, pertuzumab, tucatinib, lapatinib, larotrectinib, entrectinib, encorafenib, vemurafenib, targeted therapy, VEGF, ALK, ROS1, EGFR, HER2, NTKR, BRAF, metastatic colorectal cancer, and mCRC. The search strategy was ((((((((((((((((((((((((third-line[Title/Abstract]) OR (later-line[Title/Abstract])) OR (fruquintinib[Title/Abstract])) OR (famitinib[Title/Abstract])) OR (bevacizumab[Title/Abstract])) OR (ramucirumab[Title/Abstract])) OR (cetuximab[Title/Abstract])) OR (panitumumab[Title/Abstract])) OR (trastuzumab[Title/Abstract])) OR (pertuzumab[Title/Abstract])) OR (tucatinib[Title/Abstract])) OR (lapatinib[Title/Abstract])) OR (larotrectinib[Title/Abstract])) OR (entrectinib[Title/Abstract])) OR (encorafenib[Title/Abstract])) OR (vemurafenib[Title/Abstract])) OR (targeted therapy[Title/Abstract])) OR (VEGF[Title/Abstract])) OR (ALK[Title/Abstract])) OR (ROS1[Title/Abstract])) OR (EGFR[Title/Abstract])) OR (HER2[Title/Abstract])) OR (NTKR[Title/Abstract])) OR (BRAF[Title/Abstract])) AND ((metastatic colorectal cancer[Title/Abstract]) OR (mCRC[Title/Abstract])) AND (english[Filter]). The references of related reviews and included articles were also searched to retrieve additional studies not previously identified in the initial literature search. Inclusion criteria were as follows: clinical trials or cohort studies evaluating the efficacy and safety of third-line or later-line targeted treatment of patients with mCRC and relevant outcomes regarding treatment effects and adverse events were reported or could be calculated from the available data in the citation. Exclusion criteria included conference abstracts, case reports or case series, reviews, news, and editorials.

Two independent investigators (Wen-Hui Xue and Xue-Wei Li) accomplished the literature search and conducted the process of study selection. A third author (Wen-Hui Yang) was involved if no consensus was achieved.

### Data extraction and quality assessments

The following information was extracted from each study: name of the first author, year of publication, country, study design, number of patients, age of patients, percentage of females, patient performance, targeted molecule, lines of current treatment, therapy schedule, response rate, complete response rate (ORR), partial response rate, stable disease rate, disease progression rate, disease control rate, hazard ratios (HRs) for overall survival (OS) and progression-free survival (PFS), and adverse events rate. Clinical response and disease progression were assessed according to Response Evaluation Criteria in Solid Tumors (RECIST version 1.1) (30). The Cochrane Collaboration tool was used to evaluate the risk of bias in randomized trials enrolled in this meta-analysis (31). The methodological index for non-randomized studies (MINORS) was used for single-arm studies (32).

## Statistical analysis

The R (A language and environment for statistical computing. Version 3.6.1) was used for statistical analyses. Pooled rates and HRs with their respective 95% confidence intervals (CIs) were synthesized with a random or fixed-effects model. A random-effects model was used if the *I*² value was > 50%; otherwise, a fixed-effects model was used. The Cochran Q test was used to assess heterogeneity between studies, and the *I*² statistic was used to test the magnitude of the heterogeneity. Egger’s tests were performed to evaluate the publication bias in this meta-analysis. A *p*-value less than 0.05 was considered to be of statistical significance.

## Results

### Study selection and characteristics

**Figure 1:**
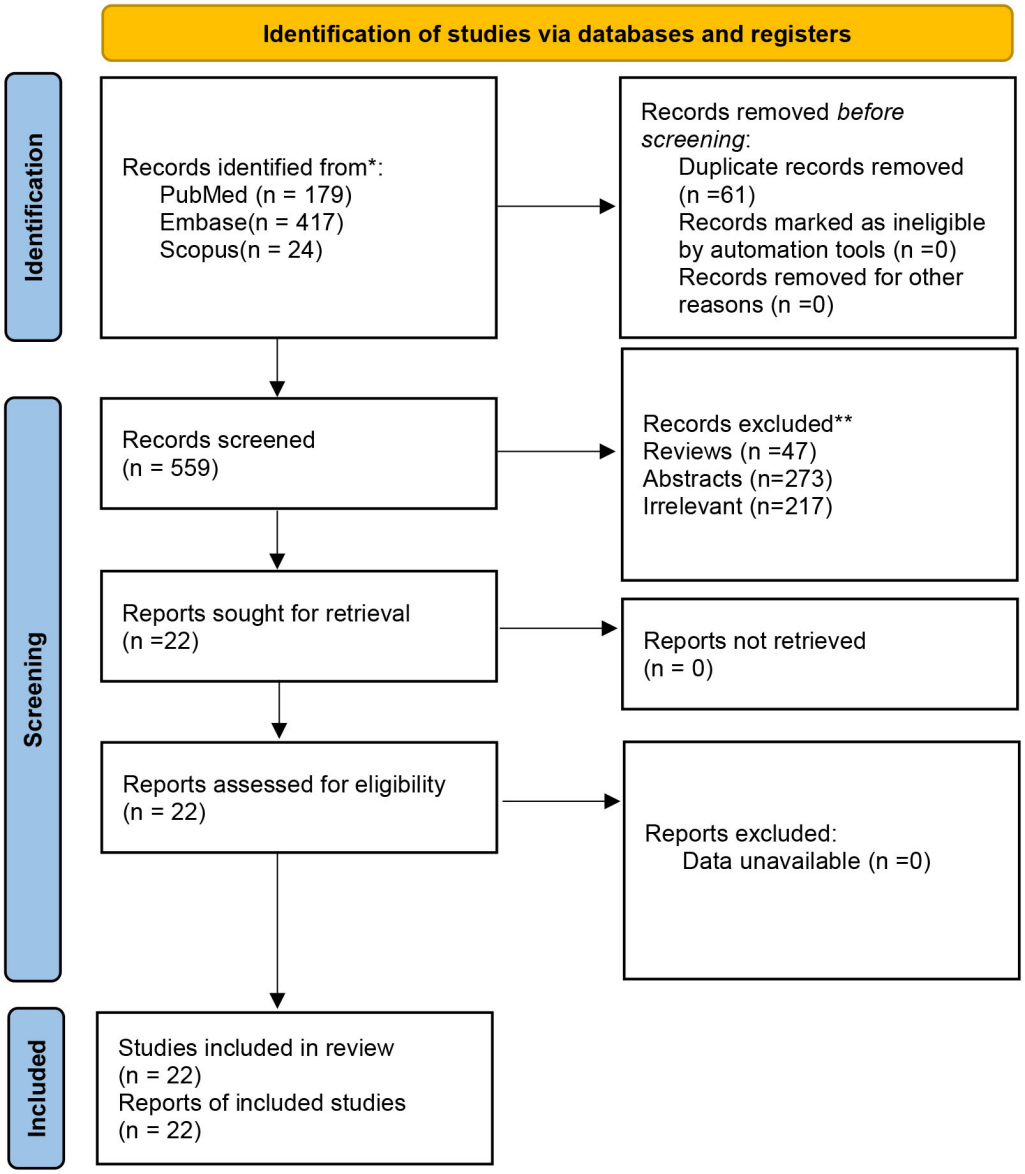
Search results and flow chart of the meta-analysis.

A total of 620 articles were identified from the databases searched. Sixty-one duplicates were eliminated, and 537 studies were excluded through an initial screening. After a full-text assessment for eligibility of the remaining 22 articles, 17 studies were eligible for inclusion in this meta-analysis, and five studies were narratively depicted. No additional studies were identified through reference screening of the included papers and relevant reviews. **Figure 1** shows details on the literature search and study selection. The enrolled 22 citations contained 1,866 patients with confirmed mCRC and reported relevant eligible outcomes for data synthesis. Twenty studies were clinical trials, and two studies were cohort studies. These studies were conducted in China, the United States, Italy, South Korea, Vietnam, France, Spain, and Japan. **Table 1** shows detailed characteristics of the included studies. The quality of included studies was rated as high based on the Cochrane Collaboration tool and the MINORS scale (**Tables 1**, **2**).

**Table 1:** Characteristics of included studies.

Author	Year	Country	Study design	No. of patient	Age (yrs)	Female (%)	ECOG-PS	Targets	Lines of treatment	Treatment schedule	Control	Tumor molecular pathology	MINORS scores
Akiyoshi (28)	2017	Japan	Phase 2	43	62 (32 - 75)	42	0 or 1	EGFR	Third	Panitumumab was administered at a dose of 6 mg/kg and irinotecan at a dose of either 150 mg/m^2^ or the tolerated irinotecan dose during prior treatment.	NA	KRAS wild type	14
Andre (23)	2012	France	Single-arm, multi-centered phase 2 study	65	62 (34 -84)	40	0 or 1 or 2	EGFR	Third	Panitumumab at a dose of 6 mg/kg on day 1 was administered as a 60-min intravenous infusion, just before the administration of irinotecan 180 mg/m^2^ in 90 min on day 1 of each fortnightly cycle (cycles are every 14 days).	NA	KRAS wild type	15
Bai (24)	2015	China	Cohort study	19	59 (48 - 72)	47	0 or 1 or 2	EGFR	Third	Cetuximab was infused at a first dose of 400 mg/m^2^ and then at 250 mg/m^2^ every week.	NA	NR	14
Chen (33)	2019	China	Single-arm phase 2 study	26	57 (28 - 75)	63	0 or 1 or 2	VEGF	Third or later	Apatinib-500 milligrams (mg) per flat dose, 28-day cycle	NA	NR	15
Chi (34)	2021	China	A Double-Blinded, Placebo-Controlled, Randomized Phase 3 Trial	419	56	36	0 or 1	EGFR	Third or later	oral anlotinib (12 mg/day; days 1–14; 21 days per cycle)	Placebo	NR	NA
Cremolini (35)	2019	Italy	Phase 2 Single-Arm	28	69 (45 - 79)	32	0 or 1	EGFR	Third	Biweekly cetuximab, 500 mg/m^2^, plus irinotecan, 180 mg/m^2^.	NA	RAS and BRAF wild type	14
Doebele (17)	2020	South Korea, Spain, USA	Phase 1	4	NR	NR	0 or 1 or 2	ROS1 and NTRK	NR	Entrectinib orally at a dose of at least 600 mg once per day	NA	NR	13
Eng (36)	2019	USA	Phase 3	75	59 (52 - 66)	57	0 or 1	VEGF	Third	Regorafenib 160 mg (group C) was given orally once daily on days 1–21 of a 28-day cycle.	NA	NR	14
Gebbia (21)	2006	Italy	Retrospective study	60	62 (37 - 81)	42	1 or 2	EGFR	Third or later	Weekly irinotecan 120 mg/m^2^ as a 1h intravenous infusion and cetuximab 400 mg/m^2^ infused over 2h as the initial dose and 250 mg/m^2^ infused over 1h for the subsequent administrations	NA	NR	13
Hong (18)	2020	USA	Phase 1	8	NR	NR	NR	NTRK	NR	Larotrectinib was administered orally (capsule or liquid formulation), continuously, on a 28-day schedule	NA	TRK fusion-positive	14
Hainsworth (19)	2018	USA	Phase 2a	37	NR	NR	0, 1, or 2	HER2	NR	Trastuzumab (8 mg/kg IV loading dose, then 6 mg/kg IV every 3 weeks) plus pertuzumab (840 mg IV loading dose, then 420 mg IV every 3 weeks)	NA	NR	14
Kopetz (37)	2015	NR	Phase 2	21	65 (38 - 91)	48	0 or 1	BRAF	Third or later	Vemurafenib was provided in microprecipitated bulk powder formulation as 240-mg film-coated tablets, dosed at the previously determined maximum-tolerated dose of 960 mg orally twice a day, and administered continuously in 28-day cycles.	NA	BRAF-mutated	13
Li (38)	2015	China, South Korea, Taiwan, and Vietnam	Randomised, double-blind, placebo-controlled, phase 3 trial	204	58 (50 - 66)	42	0 or 1	VEGF	Third or later	Patients received regorafenib 160 mg orally once daily on days 1–21 of each 28-day cycle	Placebo	NR	NA
Li (25)	2018	China	Randomised, double-blind, placebo-controlled, phase 3 trial	416	55 (23 - 75)	39	0 or 1	VEGF	Third or later	Fruquintinib, 5 mg orally, once daily for 21 days, followed by 7 days off in 28-day cycles	Placebo	NR	NA
Masuishi (39)	2020	Japan	Phase 2	34	65 (41 - 80)	32	0 or 1	EGFR	Third	Patients received 150 mg/m^2^ irinotecan intravenously every 2 weeks. Cetuximab was administered as a 2h intravenous infusion at a loading dose of 400 mg/m^2^, followed by weekly 1h infusions of 250 mg/m^2^.	NA	KRAS wild type	14
Osumi (40)	2018	Japan	Phase 2	40	59 (31 - 72)	65	0 or 1	EGFR	Third	C‐mab was initially given at a dose of 500 mg/m^2^ as a 2h infusion followed by biweekly dose of 500 mg/m^2^ as a 1h infusion. CPT‐11 was given at a dose of 150 mg/m^2^ biweekly.	NA	NR	14
Sartore-Bianchi (41)	2016	Italy	Phase 2	27	62 (50 - 68)	15	0 or 1	HER2	Third or later	Trastuzumab was given intravenously at a 4 mg/kg loading dose, then at 2 mg/kg once per week, and lapatinib was given orally at 1000 mg per day in 21-day treatment cycles.	NA	KRAS codon 12/13 wild type	14
Vincenzi (22)	2006	Italy	Phase 2	55	63 (27 - 79)	53	0 or 1 or 2	EGFR	Third	Cetuximab was given at an initial dose of 400 mg/m^2^, followed by weekly infusions of 250 mg/m^2^. Irinotecan was administered weekly at the dose of 90 mg/m^2^.	NA	NR	15
Xu (27)	2017a	China	Phase 2	71	50 (25 - 69)	40	0 or 1	VEGF	Third or later	Fruquintinib plus best supportive care	Placebo plus best supportive care	NR	NA
Xu (16)	2017b	China	double-blinded, placebo-controlled, phase 2	154	55 (24 - 71)	42	0 or 1	VEGF	Third or later	Patients were treated with 25-mg oral famitinib	Placebo	NR	NA
Yoshida (26)	2016	Japan	Phase 2	28	68 (38 - 78)	32	0 or 1 or 2	VEGF	Third	Bevacizumab was given intravenously every 2 weeks, and S-1 was administered orally on days 1–28 of a 42-day cycle.	NA	mutated KRAS	14
Yoshida (42)	2020	Japan	Single-arm, multi-centered phase 2 study	32	67 (45 - 78)	37.5	0 or 1	VEGF	Third or later	TAS-102 (35 mg/m^2^) was given orally twice daily on days 1–5 and 8–12 in a 4-week cycle, and bevacizumab (5 mg/kg) was administered by intravenous infusion every 2 weeks.	NA	NR	16

**Table 2:** Quality evaluation for Cochrane tool.

Study	Random sequence generation (selection bias)	Allocation concealment (selection bias)	Blinding of participants and peraonnel (performance bias)	Blinding of outcome assessment (detection bias)	Incomplete outcome data (attrition bias)	Selective reporting (reporting bias)	Other bias	Total quality scores
Chi, 2021 (34)	*	*	*	*	*	*	*	7
Li, 2015 (25)	*	*	*	*	*	*	*	7
Li, 2018 (38)	*	*	*	*	*	*	*	7
Xu, 2017a (27)	*	*	*	*	*	*	*	7
Xu, 2017b (16)	*	*	*	*	*	*	*	7

Each * equals 1 point.

### Treatment response

Nine studies assessed the efficacy of EGFR inhibitors monotherapy or combining chemotherapy as third-line or later-line treatment for mCRC. The other eight studies evaluated the efficacy of VEGF antibodies in treating mCRC. The pooled ORRs for monotherapy and combined therapy were 4% (95% CI: 3%, 5%) and 20% (95% CI: 11%, 29%). In the subgroup analysis of molecule targets, the pooled ORRs for VEGF and EGFR inhibitors were 4% (95% CI: 2%, 5%) and 19% (95% CI: 10%, 27%). The pooled disease progression rates for monotherapy and combined therapy were 53% (95% CI: 25%, 80%) and 34% (95% CI: 28%, 40%), respectively. The respective pooled disease progression rates for VEGF and EGFR inhibitors were 46% (95% CI: 20%, 72%) and 36% (95% CI: 29%, 43%). Concerning stable disease rates, the pooled rates for monotherapy and combined therapy were 49% (95% CI: 34%, 64%) and 43% (95% CI: 34%, 51%), and the pooled rates for VEGF and EGFR inhibitors were 57% (95% CI: 44%, 69%) and 37% (95% CI: 31%, 42%). The pooled disease control rates for monotherapy and combined therapy were 62% (95% CI: 50%, 74%) and 61% (95% CI: 54%, 68%), respectively. The pooled disease control rates for VEGF and EGFR inhibitors were 59% (95% CI: 50%, 68%) and 62% (95% CI: 54%, 71%) (**Table 3**).

**Table 3:** Subgroup analysis of treatment responses.

Treatment responses	VEGF inhibitors		EGFR inhibitors		Monotherapy		Combined therapy	
	Pooled rate	95% CI	Pooled rate	95% CI	Pooled rate	95% CI	Pooled rate	95% CI
Overall response	4%	(2%, 5%)	19%	(10%, 27%)	4%	(3%, 5%)	20%	(11%, 29%)
Disease progression	46%	(20%, 72%)	36%	(29%, 43%)	53%	(25%, 80%)	34%	(28%, 40%)
Stable disease	57%	(44%, 69%)	37%	(31%, 42%)	49%	(34%, 64%)	43%	(34%, 51%)
Disease control	59%	(50%, 68%)	62%	(54%, 71%)	62%	(50%, 74%)	61%	(54%, 68%)

EGFR, epidermal growth factor receptor; VEGF, vascular endothelial growth factor; CI, confidence interval.

The efficacy of BRAF inhibitor monotherapy for mCRC is not promising, with 0% to 5% ORRs (37). The anti-HER2 antibody trastuzumab and the dual EGFR/HER2 kinase inhibitor lapatinib were used in a phase 2 trial performed at four Italian academic cancer centers; the results were as follows: ORR of 30%, DCR of 74%, with 22% of Grade 3 toxicity (41). In addition, results of the MyPathway Study revealed that trastuzumab plus pertuzumab showed an ORR of 38% (95% CI: 23% to 55%) in 37 mCRC patients (19). In the study of Hong et al., four in eight patients with TRK fusion-positive colon cancer demonstrated a response to larotrectinib with a median response duration of 3.7 months (18). Doebele et al. reported that one in four patients with CRC responded to entrectinib, an ROS1 and NTRK inhibitor (17).

### Survival

**Figure 2:**
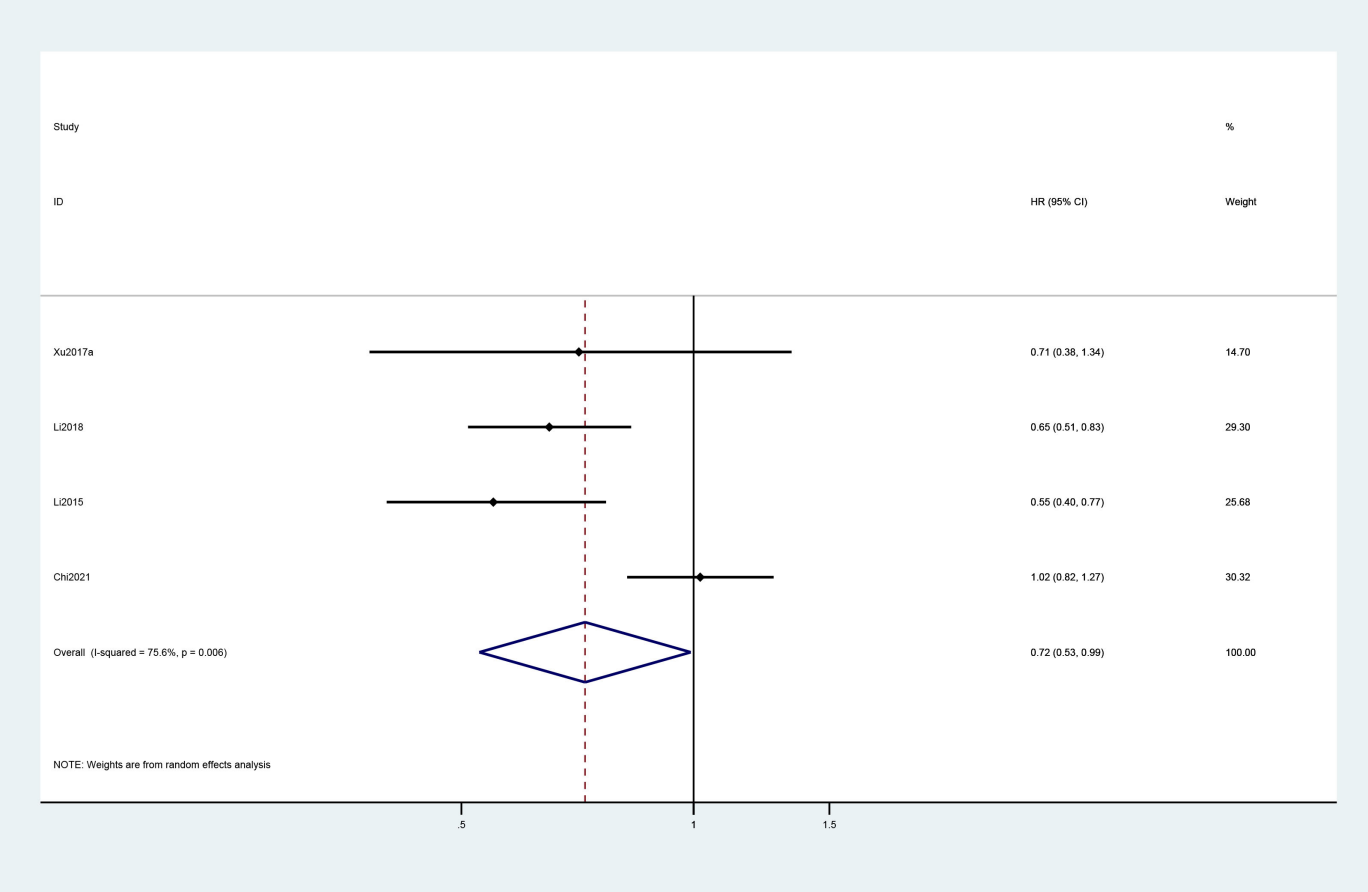
Forest plot of HR for overall survival in included studies.

**Figure 3:**
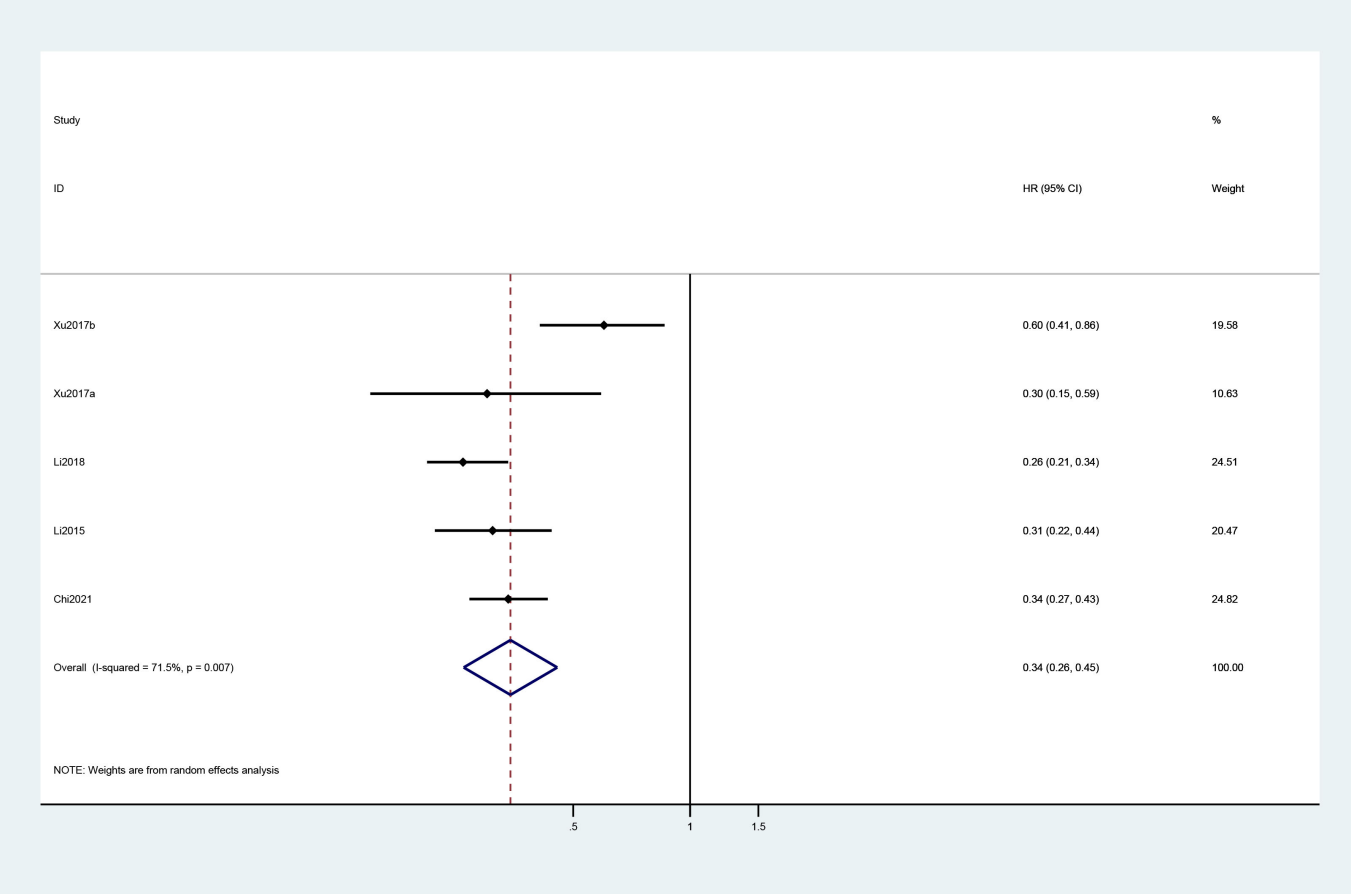
Forest plot of HR for progression-free survival in included studies.

A total of four studies reported the Kaplan–Meier estimates of overall survival in the treatment and control groups. The pooled HR was 0.72 (95% CI: 0.53, 0.99) (**Figure 2**). For PFS, the pooled HR of five trials was 0.34 (95% CI: 0.26, 0.45) (**Figure 3**).

### Adverse events

Hematological adverse events were the most frequently reported in included studies. The occurrence rates of anemia for VEGF and EGFR inhibitors were 26% (95% CI: 7%, 44%) and 42% (95% CI: 3%, 87%). The pooled occurrence rates of leucopenia for VEGF and EGFR inhibitors were 36% (95% CI: 9%, 63%) and 33% (95% CI: 6%, 60%). With regard to neutropenia, pooled occurrence rates for VEGF and EGFR inhibitors were 34% (95% CI: 9%, 60%) and 47% (95% CI: 24%, 71%). The occurrence rates of thrombocytopenia for VEGF and EGFR inhibitors were 25% (95% CI: 14%, 36%) and 18% (95% CI: 12%, 23%).

### Publication bias

*P*-values of Egger’s tests for publication bias were < 0.001, 0.129, 0.001, 0.052, 0.588, 0.622, 0.078 in the pooled analyses of overall response, stable disease, disease progression, disease control, HR for OS, HR for PFS, and adverse events, respectively.

## Discussion

CRC is one of the most important tumors, with high incidence and mortality rates worldwide (43). Many patients are diagnosed at the metastatic stage of the disease; for these patients, treatment is mainly based on chemotherapy (44). Maintaining the quality of life is the primary goal and urgent need of mCRC patients undergoing third-line or later-line treatment (8). However, few insights are gained to guide the selection and sequencing of treatments for these patients (10, 14). Recently, prolonged OS in patients with mCRC has been observed through targeted therapies, such as antibodies against EGFR and VEGF (44).

In the meta-analysis, 17 published articles containing 1,769 patients with diagnosed mCRC and treated with targeted therapies were included. This meta-analysis showed that the pooled ORRs for VEGF and EGFR inhibitors were 4% and 19% in the third-line or later-line treatment of mCRC. Targeted therapy combined with chemotherapy demonstrated favorable ORR and disease control rate with less disease progression than target monotherapy. The results corroborated the findings from previous clinical trials. Furthermore, targeted therapy revealed increased OS and PFS; the goal of the third-line or later-line treatment is to prolong survival and prevent tumor progression without affecting the quality of life. The molecular type of mCRC in included studies was not specified. It is reported that the benefit in PFS and OS was observed only in the KRAS wild-type patients for both cetuximab and panitumumab (45, 46). Moreover, the NCCN clinical practice guideline recommended that regorafenib could be utilized in fit patients with the refractory disease after standard chemotherapy including fluoropyrimidine, oxaliplatin, and irinotecan and anti-VEGF or anti-EGFR therapies (RAS wild type) (47).

However, hematological adverse events, including anemia, neutropenia, leucopenia, and thrombocytopenia, were commonly observed in included studies. In addition, the evidence on clinical trials of other targeted therapies, namely, BRAF inhibitors, HER2 inhibitors, anti-NTRK agents, and ROS1 inhibitors, was limited, so we could not pool these outcomes. Therapies with HER2, NTRK, and ROS1 blockade have shown significant antitumor activity, and more well-designed clinical trials are needed to verify the efficacy and safety of these agents. It is recommended in HER2-positive patients with mCRC, treatment with HER2 dual blockade is optionally recommended, especially in RAS WT tumors (48).

This meta-analysis was conducted at the population level, because individual patient data cannot be obtained. In the current study, a comprehensive literature search in English was performed to increase the probability of obtaining all relevant included studies. Data extraction was conducted by two independent reviewers using a pre-designed form. In addition, we assessed the quality of enrolled studies using the Cochrane Collaboration tool and the MINORS scale. The quality of included studies was rated as high. We assessed the heterogeneity between the studies. Results showed significant heterogeneities in the analyses of OS and PFS. The heterogeneity may be attributed to differences in patient characteristics, study design, drug compliance, prior lines of therapy in each study, and other relevant factors. Due to heterogeneity between third-line or later-line treatment regimens, it is difficult for us to determine the specific subgroups, and therefore subgroup analysis based on regimens was not performed. Meta-regression was not performed due to a limited number of studies in each subgroup. Furthermore, the findings of Egger’s tests indicated that publication bias might not be neglected in analyzing several indicators. Albeit with the heterogeneity and publication bias in included studies and limitations of this meta-analysis, the results may provide evidence-based information on the efficacy and safety of third-line or later-line target therapy for patients with mCRC.

Based on the outcomes of this meta-analysis, we may conclude that targeted therapies, including VEGF and EGFR inhibitors, showed promising clinical response rates and prolonged survival in the treatment of mCRC patients with progression after first- and second-line therapy. Targeted therapy for mCRC patients with biomarker selection may improve marginal prognosis but is unlikely to change the treatment pattern of most patients significantly. Incidences of hematological adverse events were durable and acceptable. However, the pathogenesis of these adverse events remains poorly understood (49). Personalized treatment or combined therapy was recommended based on the feature of mCRC. It is expected that well-designed clinical trials, as well as real-world studies, should be conducted to address issues on the evaluation of efficacy and safety of VEGF and EGFR inhibitors and other targets in the treatment of mCRC. Very preliminary evidence was found regarding the targets of HER2, NTRK, and BRAF. Further studies are needed to investigate if such targets may perform an essential role as VEGF and EGFR in the later line management of mCRC.

## Data availability statement

The original contributions presented in the study are included in the article/supplementary material. Further inquiries can be directed to the corresponding authors.

## Author contributions

W-HY and JX conceived and designed this study. W-HX, X-WL, Y-QD, NW, B-BP, and X-YM were responsible for the collection, extraction, and analysis of the data. W-HX were responsible for writing the paper. W-HY and JX performed the quality evaluation and completed data analysis. W-HY polished the English language. All authors and participants reviewed the paper. All authors contributed to the article and approved the submitted version.
